# Complexities in Genetics of Psoriatic Arthritis

**DOI:** 10.1007/s11926-020-0886-x

**Published:** 2020-03-12

**Authors:** Sara Rahmati, Lam Tsoi, Darren O’Rielly, Vinod Chandran, Proton Rahman

**Affiliations:** 1 Department of Medicine, Faculty of Medicine, Memorial University, St. John’s, Newfoundland and Labrador A1B 3X9 Canada; 2 Krembil Research Institute, University Health Network, Toronto, Ontario M5S 1A8 Canada; 3 Department of Computational Medicine & Bioinformatics, Department of Biostatistics, University of Michigan, Ann Arbor, MI 48109 USA; 4 Institute of Medical Science, University of Toronto, Toronto, Ontario M5S 1A8 Canada; 5 Department of Laboratory Medicine and Pathobiology, University of Toronto, Toronto, Ontario M5S 1A8 Canada; 6 Department of Medicine, Division of Rheumatology, University of Toronto, Toronto, Ontario M5S 1A8 Canada

**Keywords:** Psoriasis, Psoriatic arthritis, Genetics, Genome wide association scans, Integrative medicine, Functional pathways

## Abstract

**Purpose of the Review:**

To provide a general overview and current challenges regarding the genetics of psoriatic disease. With the use of integrative medicine, multiple candidate loci identified to date in psoriatic disease will be annotated, summarized, and visualized. Recent studies reporting differences in genetic architecture between psoriatic arthritis and cutaneous-only psoriasis will be highlighted.

**Recent Findings:**

Focusing on functional pathways that connect previously identified genetic variants can increase our understanding of psoriatic diseases. The genetic architecture differs between psoriatic arthritis and cutaneous-only psoriasis with arthritis-specific signals in linkage disequilibrium independent of the published psoriasis signals.

**Summary:**

Integrative medicine is helpful in understanding cellular mechanisms of psoriatic diseases. Careful selection of the psoriatic disease cohort has translated into mechanistic differences among psoriatic arthritis and cutaneous psoriasis.

**Electronic supplementary material:**

The online version of this article (10.1007/s11926-020-0886-x) contains supplementary material, which is available to authorized users.

## Introduction

Psoriasis is a common, inflammatory, hyperproliferative cutaneous disorder with an estimated prevalence of approximately 3% in the US adult population [1]. Psoriatic arthritis (PsA) is a distinctive inflammatory arthritis that occurs in approximately 20 to 30% of psoriasis patients [2, 3], making inflammatory arthritis the most common immune mediated extra-cutaneous manifestation of psoriasis. Psoriasis and PsA are multifactorial in origin as pathogenesis is influenced by genetic factors and environmental triggers (e.g., streptococcal infections and trauma) leading to dysregulation of key immunological pathways involving Th1-, Th2-, and Th17-based cytokines [2, 3]. Using candidate gene studies involving *HLA* alleles and large-scale dense SNP-based association studies, currently over 70 genome-wide statistically significant candidate loci have been associated with psoriatic disease, a subset of which is also associated with PsA [4, 5, 6•, 7]. However, not all these loci have been consistently replicated, and the effect size of any given genetic loci outside the MHC region is modest (odds ratio < 1.2). Also, there is incomplete or limited information on the proposed function and relevance of some genes implicated in psoriatic disease pathology. At present, there is limited clinical utility in prospectively determining genetic variants for diagnosis, prognosis, or management of psoriatic disease.

Elucidating the genetic determinants of PsA has been a greater challenge given that there are only a handful of genes specific to inflammatory arthritis that are not associated with psoriasis as most of the candidate loci associated with PsA are also associated with psoriasis. As recent comprehensive reviews have been published chronicling the long list of genetic loci associated with psoriatic disease [5, 7–9], the purpose of this review is to briefly summarize the key genetic elements of PsA, highlighting key challenges encountered in identifying disease-related genes in PsA disease pathology. Given the extensive list of genetic loci associated with psoriatic disease, we will attempt to systematically collate and synthesize genes into their respective locations, implicated pathways, overlapping functions, as well as associations noted in psoriasis and PsA. Finally, we will review recent studies that compared PsA and cutaneous-only psoriasis (PsC) to determine differences in the genetic architecture between the two entities.

## Phenotypic Challenges: Heterogeneous Cutaneous and Articular Involvement

Psoriatic disease is a heterogenous entity, encompassing several types of psoriasis, assorted array of extra-cutaneous features, as well as co-morbidities. Regarding the articular manifestations, synovitis, enthesitis, dactylitis, and spondylitis can occur, and each phenotype may be attributed to a unique genetic contribution [2, 3]. These manifestations of PsA are not mutually exclusive but often coexist. Given these challenges, genetic studies in psoriatic disease predominantly focus on type I psoriasis vulgaris (age of psoriasis onset occurs < 40 years), and the inflammatory arthritis is predominantly oligo or the polyarticular variant that may or may not be associated with concomitant spondylitis, enthesitis, or dactylitis [8, 9]. Given the large number of unique phenotypes that can be present, clinical heterogeneity will likely be reflected by more complex genetic architecture. Careful phenotyping of patients, analyzing large cohorts, and leveraging the latest advances of artificial intelligence may be required to identify clinically meaningful genetic clusters involved in psoriatic disease.

A second challenge, particularly for case-control studies, is the definition of case and control when attempting to identify PsA-specific genes in psoriasis patients. Comparison of psoriatic disease (cutaneous and vulgaris psoriasis) from unaffected controls is relatively straightforward as is deciphering differences between PsA from healthy controls. However, identifying susceptibility factors of inflammatory arthritis among patients with psoriasis is a much greater challenge. It is difficult to confidently distinguish PsC from PsA patients, as current patients with PsC may develop PsA at a later time point. The second scenario relates more to dermatology-led initiatives where inflammatory arthritis may actually coexist but is not recognized as a systematic clinical, laboratory, or imaging assessment was not performed. In an attempt to overcome these challenges, investigators are including patients with at least 7 to 10 years of psoriasis and no musculoskeletal (MSK) symptoms and collaborating with rheumatologists to ensure no inflammatory arthritis is present at time of enrollment. The final issue regarding the PsA phenotype is the overlap with psoriasis as up to 85% of PsA patients have psoriasis at diagnosis, and most of the remaining patients will develop psoriasis with time [2, 3]. This overlap is unavoidable as PsA is an entity that is associated with psoriasis.

## Estimating the Genetic Burden of Disease: Conflicting Heritability Scores Based on Methodology

The genetic contribution for a complex multifactorial disease is usually gathered from multiple lines of evidence [4, 6•]. Epidemiological studies are a well-recognized method to assess familial aggregation of complex diseases. Clinic- or population-based epidemiological studies suggest a strong genetic contribution of PsA, as the relative risk of an affected first-degree relative is 30- to 55-fold higher in PsA probands as compared with siblings of patients unaffected with PsA [10]. In contrast with psoriasis, the heritability estimated from epidemiological studies has not been validated from twin studies as no large PsA twin studies exist. Twin-based validation is important as heritability based solely on epidemiological approach is very sensitive to disease prevalence, and underestimating disease prevalence can overestimate heritability. A recent secondary analysis of genome-wide association (GWA) studies suggests that heritability of PsC and PsA may be similar [11]. That is, analysis is preliminary and dependent on the methods used to estimate heritability; further work is warranted to define the heritability of PsA as compared with psoriasis.

## Role of the Environment: a Missing Link Not Systematically Captured

PsA is clearly a multifactorial disease, and environmental factors are crucial to understand disease pathogenesis. That being said, systematic evaluations of environmental determinants are sparse. Stress can flare psoriasis, and there is a growing body of evidence emerging in neuro-immunology regarding the role of mental stress and the immune system [2, 3••]. More traditional risk factors include mechanical stress and selected infections [2, 3••, 4, 6]. There is now substantive evidence implicating a role of the microbiota in psoriatic disease [12]. These environmental determinants are beyond the scope of this article, but it is important to acknowledge that identification of these triggers is necessary to interrogate the gene/environmental factors in a systematic fashion. Without careful documentation of the type, duration, and magnitude of environmental exposure, it is not possible to accurately investigate the role of the environment is PsA pathology.

## Linkage Studies: Only of Historical Significance?

Linkage studies, whether based on the recombinant principle or excessive allele sharing, are family-based studies using parametric and nonparametric analysis, respectively, to identify high priority candidate loci. Most linkage studies in psoriatic disease have focused on psoriasis vulgaris to identify susceptibility regions across the genome. In total, nine areas of interest designated *PSORS1* to *PSORS9* have been identified, of which only three were replicated [13]. Searching for candidate genes within these regions has culminated in very few validated candidate genes other than PSORS1 where *HLA-Cw6* and *CDSN* reside, *CARD14* in PSORS2 and *LCE3B* and *LCE3C* in *PSORS4* (as reviewed by Capon [13]). *HLA-Cw6* was implicated well before linkage studies were initiated [14]. *CARD14* is unique in that it appeared to segregate in an autosomal dominant fashion, thus amenable to identification using linkage analysis [15]. The utility and enthusiasm of linkage studies have completely faded for polygenic multifactorial disease and are now mostly of historical significance. That being said, there is still much to be learned from multi-generational multiplex families, particularly for rare variant detection using next-generation (whole genome or exome) sequencing.

## Candidate Gene Studies: Multiple Robust *HLA* Associations

Case-control studies of candidate genes have been the most traditional approach for gene identification in PsA. As the proposed function of *HLA* alleles within the adaptive immune system is peptide binding and presentation to the T cell, the MHC region continues to be the most thoroughly investigated region within the genome for psoriatic disease. The most robust association is with HLA-C*06:02. The magnitude of the association of HLA-C*06:02 differs significantly between PsC and PsA (much stronger for PsC than PsA when compared with unaffected controls) suggesting that the two entities are genetically heterogeneous as they likely do not entirely arise from the action of the same *HLA* genes. Four alleles from the *HLA-B* locus are much more likely to be associated with PsA (HLA-B*27:05, HLA-B*39:01, HLA-B*38:01, and HLA-B*08:01), whereas HLA-B*44:02 is likely not associated with disease [8, 9]. These alleles are likely in linkage disequilibrium with ancestral haplotypes. Furthermore, the peptide-binding properties of HLA-B*44:02 differ from HLA-B*27:05 and HLA-B*39:01 [16, 17], supporting the concept that particular peptides preferentially bind to select HLA molecules, resulting in disease susceptibility or prevention.

The HLA associations exhibit the strongest genotype/phenotype correlation. The presence of HLA-Cw*06:02 is associated with earlier onset of psoriasis, a delayed interval between skin and joint disease and less peripherally damaged joints (5). HLA-B*27:05 is much more prevalent in PsA spondylitis, symmetric sacroiliitis, enthesitis, and dactylitis (5). The presence of HLA-B*27:05 and HLA-B*39:01 is associated with less time lag between psoriasis and PsA [18–20••]. HLA-B*08:01 is associated with more peripheral arthritis, joint deformity, and ankylosis and asymmetric sacroiliitis [18–20••]. Overall, HLA-C*06:02 and HLA-B*44:02/03 are associated with a milder arthritis phenotype [21]. HLA typing is not routinely used in the clinic with the possible exception of PsA sine psoriasis. However, if relative risk scores are modeled strategically, it is conceivable that these associations may help predict diagnosis or potentially subset disease.

## GWA Studies: Analysis and Deciphering of Multiple Susceptibility Loci

**Fig. 1:**
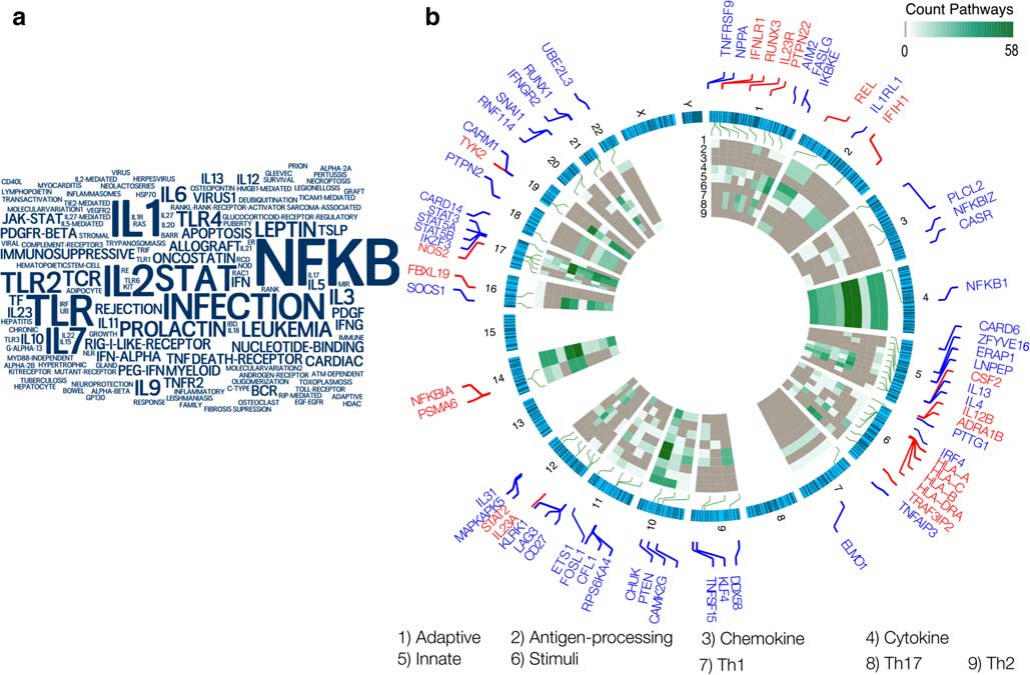
(**a**)Term enrichment analysis of enriched pathways with genes related to genomic variations linked with psoriasis. Pathway and term enrichment analysis are performed using pathDIP (version 4; literature-curated pathways), and word cloud is generated using “wordle.net.” (**b**) Summary of immune system pathway classes and chromosomal location of genomic variations linked with psoriasis. The outside circle shows chromosomal location of 69 psoriasis genes which belong to the enriched immune system pathways. Genes in red font highlight variations significantly related to PsA, while blue genes have been linked to any psoriatic disease. Enriched immune system pathway are grouped in nine major classes, and the circular heatmap shows the number of pathways from each class that each gene belongs to. There are only a few genes in crosstalk of all nine pathway classes. None of these genes are located on chromosomes with high numbers of immune system genes in psoriasis. Plot is generated using OmicCircos R package version 1.18.0

The greatest progress regarding identification of statistically significant loci has been through large-scale SNP-based arrays. Collection of large psoriatic cohorts, genotyping with extensive SNP-based arrays and meta-analyses and other methodological improvements to interrogate large datasets, has translated into insightful genetic associations in psoriatic diseases. However, disease-related cellular mechanisms underlying these genetic variations are not currently understood [16, 21–24]. To this end, we used an integrative approach to annotate, summarize, and visualize the relevance of genes identified through multiple GWA studies in psoriatic disease with signaling pathways and their corresponding chromosomal locations. We obtained a list of 133 genes with variations significantly associated with psoriatic disease from a recent review by Stuart et al. [5, 7] and used pathway data integration portal (pathDIP, version 4[25]), a comprehensive integrated database for annotation and enrichment analysis of genes, to investigate pathways of relevance to psoriatic disease pathology. Overall, 105 (of out of 133) genes are annotated with 1366 (out of 5380) literature-curated pathways (Supplementary Table 1A) of which 416 pathways show significant enrichment (*q*-value < 0.05) and cover 83 of these genes (Supplementary Table 1B). Summarizing pathways through the term enrichment analysis tool in pathDIP highlighted key terms mostly relevant to the immune system (Fig. 1a), and therefore, we focused on immune system pathways. First, using the key terms and *search pathway* tool in pathDIP, we identified 69 (out of 105) genes annotated with 244 immune system pathways. We manually curated pathways and annotated them with nine (possibly overlapping) major classes: adaptive, antigen-processing, chemokines, cytokines, innate, stimulus, Th1, Th2, and Th17 (Supplementary Tables 2–3). Next, each gene was annotated with the number of pathways assigned to each of the nine major classes (ranging between 58 and 0 pathways). The chromosomal location and number of pathways of each class for each of the 69 genes and whether it is significantly associated with PsA are illustrated (Fig. 1b). The outermost circle represents the 22 chromosomes and reveals that chromosomes 1, 5, 6, and 12 have the highest number of immune system gene associations with psoriasis, while chromosomes 8, 13, and 15 are depleted of variations of immune system genes relevant to psoriatic disease. Information on the *X* and *Y* chromosomes is lacking due to the absence of association analysis on the sex chromosomes (sex information is missing for some cohorts in the meta-analysis). Among chromosomes with the most aberrations, chromosome 6 (five genes) and chromosome 1 (four genes) hold the maximum number of genes associated with PsA (Fig. 1b; labeled in red). In addition, the circular heatmap illustrates the number of pathways in each of the predefined nine immune system pathway classes. *NFKB1*, *CHUK*, *STAT3*, *NFKBIA*, *STAT5A*, and *STAT5B* are annotated with 110, 99, 90, 93, 71, and 66 immune system pathways, respectively, and cover all nine major immune classes, suggesting their important role in connecting different classes of immune system pathways. However, *NFKBIA* is the only gene whose variation association with PsA has reached statistical significance. Intriguingly, all of these six genes are located on chromosomes containing small numbers of immune system genes linked with psoriasis, suggesting that chromosomal regions most populated with psoriasis-related variations may not represent the best indicator of importance in disrupting cellular pathways related to disease pathology. For example, chromosomes 10 and 14 are among chromosomal regions with the smallest number of psoriasis genes, even though two of their genes demonstrate maximum centrality in connecting major classes of immune system pathways. Furthermore, among the nine immune classes, cytokines, innate, and adaptive annotate the greatest number of psoriasis genes (52, 44, and 39, respectively), while chemokines and antigen-presentation annotate only 11 and 13 genes, respectively.

In addition to the 243 immune system pathways summarized (Fig. 1b), 172 pathways are enriched with psoriasis genes (Supplementary Table 1A) which are not part of the nine immune system pathway classes. We used titles of these 172 pathways to define seven (possibly overlapping) classes of pathway key terms: tightly-immune-related processes, immune-related diseases, metabolism/metabolic-disease, apoptosis, osteogenesis, skin/skin-diseases, and other processes. We identified 233 pathways containing key terms in these seven classes (Supplementary Table 1B). The number of pathways annotated with each category is 47, 28, 39, 16, 10, 10, and 124, respectively, and they annotate 76 (out of 133) psoriasis genes (Supplementary Table 4). The frequency of all key terms in the titles of these 233 pathways is also provided (Supplementary Table 5). Many of these key terms have been previously linked to pathogenesis of psoriatic diseases, such as metabolic pathways [26, 27], *NOTCH* [28–30], *WNT* [31–33••], *P38/MAPK* [34–36], and apoptosis [37–39]. TNF-alpha inhibitors, directly or indirectly, target G protein-coupled receptors (GPCRs) which are among highly frequent non-immune system pathway terms in our analysis. The importance of investigating GPCR pathways in rheumatoid and psoriatic arthritis has been previously highlighted in the literature [40].

Our analysis identified 62 genes being shared between the nine classes of immune system pathways and the genes in the additional seven pathway classes. However, the removal of the 69 immune system pathway genes from the list of 105 genes with available annotations in pathDIP (Supplementary Table 1B), surprisingly, returned zero enriched pathways, suggesting a central role of the 69 highlighted genes in crosstalk between immune- and non-immune system enriched pathways. Interestingly, the five non-immune system genes whose variations are significantly associated with PsA (i.e., *ANXA6*, *HSD3B7*, *KCNH7*, *P4HA2*, *TNIP1*) are enriched in RAC1/PAK1/P38/MMP2, synthesis of bile acids/salts and prostaglandin, metabolism of bile acids, arginine and lipids, and biosynthesis of steroid and collagen pathways. These findings are supported through previous research that has linked many of these pathways with PsA and severe psoriasis cases [41–44].

In total, out of 133 genes in our original gene list, only 105 have pathway annotations in the literature, leaving 28 genes as pathway orphans—genes with no pathway annotation in the literature [45]. However, extended pathDIP increased overall coverage of pathway annotations to 119 genes, 106 of which are associated with immune system pathways. Extended pathways are based on the strong physical connectivity of each protein with members of each pathway, thus, annotation of an additional 37 genes with immune system pathways suggests strong physical and functional association with the immune system at the molecular level. Of the 37 genes, those associated with the highest number of immune system pathways are *TP63* and *TNIP1* with 184 and 167 pathways, respectively (Supplementary Table 6). Interestingly, the interplay between *TP63* and *NFKB* and its potential role in inflammation and genetically-mediated defects in *TNIP1* in inflammatory cells in psoriasis have been speculated in two recent reviews [46, 47].

Despite these findings, systematic annotation of genomic variations in psoriatic disease with literature-curated and extended pathways left 13 genes and loci with no pathway annotation. These 13 loci are poorly studied and searching other resources such as Uniprot [48] or Gene Ontology [49], retuned functional annotations for some of the loci covering a wide range of processes. UniProt annotates *GPR160* with the GPCR protein family, *RNF145* with ubiquitin ligase activity, *RP11* (*PRPF31*) with pre-mRNA splicing, and *ZNF365* with homologous recombination and maintaining genomic stability. Gene Ontology annotates *KLLN* with DNA binding, apoptosis, and cell-cycle and *ZNF683* with adaptive and innate immune system and T cell differentiation regulation. The remaining five loci (*C1ORF141*, *CCDC129 (ITPRID1)*, *LINC00330*, *LINC01185*, *RN7SK)* are not protein-coding genes or do not have any functional annotations in UniProt and Gene Ontology. Lack of available functional information for these loci highlights that despite all the efforts to date, understanding cellular mechanisms of psoriatic diseases is still at its early stages. In addition, focusing only on enriched pathways can ignore useful information, partly, due to incomplete data regarding disease and functional and physical relationships among genes. For example, although *LCE3A-D* have been linked with psoriatic diseases, they did not show up in our analysis as they are annotated with pathways that were not identified as pathways enriched with psoriasis genes.

Nevertheless, our integrative approach is one step closer toward understanding the systems biology of psoriatic disease. Rather than focusing on single genomic variations or chromosomal locations, our integrative approach focused on functional pathways that connect previously identified variations. Enrichment analysis of these variations, their systematic annotation with pathways and summarizing them into major pathway classes, along with integrating chromosomal locations of the involved genes, not only highlighted pathways implicated in the disease but also characterized genes that may be major role players in connecting different disrupted functional pathways. Our systematic review provides a novel perspective to study and prioritize contributors to psoriatic diseases at the molecular and functional level.

## Differences in Genetic Architecture of PsA and PsC

Driven by the elevated genetic heritability in PsA (~80 to 100%), when comparing with psoriasis in general (around 50 to 90%), multiple studies have attempted to understand the genetic differences between PsA and PsC [50, 51, 52••, 53••]. Since most PsA patients develop arthritis within 10 years after skin symptoms appear, both studies of Patrick et al. [50, 52••] and Stuart et al. [51, 53••] compared PsA with PsC (defined by psoriatic patients without the development of PsA for at least 10 years). While this approach will restrict to non-PsA patients that have many years of follow-up, it can avoid the reduction of power when comparing PsA to a group mixed with psoriatic patients that have potential to develop PsA. In Stuart et al. [51, 53••], the authors investigated 3061 PsA cases, 3110 PsC cases, and 13,670 controls from six different cohorts, including the immunochip cohort, consisting of genotype data from targeted regions. The authors devised an indirect PsA versus PsC comparison approach that can take advantage of the genetic cohort that only contains one psoriasis subtype (i.e., PsA) along with control samples, and summary statistics derived from the PsA vs control comparison were compared against those derived from the PsC vs control comparison. This effort revealed different psoriasis-associated variants with stronger effect in PsC (i.e., loci encompassing *HLA-C*, *TNFRSF9*, and *LCE3A*) or with stronger and more specific effect in PsA (i.e., loci near *IL23R* and *TNFAIP3*). The authors further revealed that PsA-specific signals are linkage disequilibrium-independent of the published psoriasis signals, highlighting that established psoriasis loci identified from GWA studies consisting majority of non-PsA individuals might have dampened the PsA-specific effect, and conditional analysis might be needed to further unravel secondary signals that are sub-phenotype specific.

A study by Patrick et al. [50, 52••] enhanced genetic analysis by taking advantage of the new “exomechip” cohort with genome-wide coverage and the new imputation reference panels to conduct psoriasis sub-phenotype association analysis. Importantly, this study was able to increase the number of genotyped samples with genome-wide coverage (i.e., 2703 PsA and 2681 PsC samples compared with the 1946 PsA and 1363 PsC samples in a study by Stuart et al. [51, 53••]). Since there is only one genome-wide significant locus differentiating PsA vs PsC samples (i.e., the *HLA-C* in MHC), the authors investigated chromatin marks that are enriched among markers showing evidence of association when comparing PsA vs PsC. The results illustrated the enrichment of regulatory regions in immune cells, potentially driven by markers in the MHC, and in a follow-up study by the same group, they further demonstrated that non-MHC markers showing evidence of association (i.e., *p < 1 × 10*^*−4*^), when comparing PsA vs PsC, are significantly and specifically enriched with H3K27ac marks for osteoblasts, and those loci overlapping with chromatin marks encompass genes participating in the Wnt signaling pathway [32, 33••]. These biologically relevant results further hint that there are other genetic loci with modest effect sizes that differentiate PsA and PsC patients, and thus larger sample size is needed to differentiate them from false-positive results. Subsequently, the study by Patrick et al. [50, 52••] implemented a machine learning approach to provide risk assessment for PsA among psoriatic patients, taking into account the expected prevalence of ~30% for psoriatic patients. Interestingly, genome-wide coverage is able to achieve higher performance, further highlighting the potential roles from non-MHC loci to characterize psoriasis subtypes. Nevertheless, the authors discussed potential limitations in clinical application due to differences in demographic and population structures of different cohorts. Consequently, an optimized model is reusing samples that can represent the best patient population structure in question.

## Conclusion

Substantive progress has been made in the genetics of psoriatic disease over the last decade primary due to SNP-based large-scale association studies, as over 70 candidate loci have reached genome-wide statistical significance. Larger cohorts with detailed clinical and imaging phenotyping will likely lead to further candidate loci, particularly for well-defined subsets of psoriatic disease. These studies will be complemented by data derived from NGS of the whole exome and/or whole genome which will identify rare variants for multiplex families. In addition, integration and computational analysis can provide insight into functional relationships among identified variations and characterize novel signaling pathways. Differentially expressed genes via transcript profiling, micro-RNA, circulating RNA, and single-cell sequencing are presently a major area of focus, and we are eagerly awaiting results from these endeavors. Furthermore, concerted efforts are directed at epigenetic sites, including DNA methylation sites and open chromatin. Finally, combining genomics with additional omics, such as proteomics, metabolomics, and microbiomes, may further strengthen proposed function of new genomic findings. Thus, we are still at the early stages of interrogating the genetic basis of psoriatic disease, and the rapid emergence of affordable high-throughput technology, coupled with advances in big data analysis and integrative medicine, will likely lead to numerous novel candidates, some of which will hopefully lead to improved diagnosis, prognosis, and therapeutic response for psoriatic disease.

## Electronic supplementary material

ESM 1 (XLSX 7073 kb)

## References

[1] Rachakonda TD, Schupp CW, Armstrong AW (2014). Psoriasis prevalence among adults in the United States. J Am Acad Dermatol.

[2] Ritchlin CT, Colbert RA, Gladman DD (2017). Psoriatic arthritis. N Engl J Med.

[3] Ritchlin CT, Colbert RA, Gladman DD (2017). Psoriatic arthritis. N. Engl. J. Med.

[4] O’Rielly DD, Rahman P (2015). Genetic, epigenetic and Pharmacogenetic aspects of psoriasis and psoriatic arthritis. Rheum Dis Clin N Am.

[5] Stuart, P. E., Tsoi, L. C, Hambro, C. A, Elder, J. T. Genetics of psoriasis. In: Fitzgerald O, Gladman D, editors. Oxford Textb. Psoriatic Arthritis. 1st Edition. 2019a. p. 35–55.

[6] O’Rielly DD, Rahman P (2015). Genetic, epigenetic and pharmacogenetic aspects of psoriasis and psoriatic arthritis. Rheum. Dis. Clin. North Am.

[7] •• Stuart, P. E, Tsoi, L. C, Hambro, C. A, Elder, J. T. Genetics of psoriasis. In: Fitzgerald O, Gladman D, editors. Oxford Textb. Psoriatic Arthritis. 1st ed. 2019b. p. 35–55. **Exhaustive reivew of psoriasis with comprehensive list of susceptibility loci in psoriasis****.**

[8] Winchester R, O’Reilly, D P R. Genetics of psoriatic arthritis. In: FitzGerard O, Gladman D, editors. Oxford Textb. Psoriatic Arthritis. 2019a. p. 57–67.

[9] • Winchester R, O’Reilly D. P R. genetics of psoriatic arthritis. In: Oxford Textb. Psoriatic arthritis. Fitzgerard O, Gladman D ed; 2019b. p. 57–67. **A very practical review of the MHC associations and non-mHC associations in psoriatic arthritis****.**

[10] Rheum Dis Rahman AP, Elder JT, Rahman P. arthritis genetic epidemiology of psoriasis and psoriatic topic collections genetic epidemiology of psoriasis and psoriatic arthritis. Ann Rheum Dis [Internet]. 2005;6464:37–39. Available from: http://ard.bmjjournals.com/cgi/content/full/64/suppl_2/ii37%0Ahttp://ard.bmjjournals.com/cgi/content/full/64/suppl_2/ii37#otherarticles%0Ahttp://ard.bmjjournals.com/cgi/content/full/64/suppl_2/ii37#BIBL 10.1136/ard.2004.030775 PMC1766868 15708933

[11] Quan L, Chandran V, Tsoi L, Nair R, Gladman DD, Elder J (2019). AB0020B quantifying differences in heritability among psoriatic arthritis (psa), cutaneous psoriasis (PSC) and psoriasis vulgaris (PSV). Ann Rheum Dis.

[12] Yan D, Issa N, Afifi L, Jeon C, Chang H-W, Liao W (2017). The role of the skin and gut microbiome in psoriatic disease. Curr Dermatol Rep.

[13] Capon F. The genetic basis of psoriasis. Int. J. Mol. Sci. [Internet]. 2017;18(12). Available from: http://www.ncbi.nlm.nih.gov/pubmed/29186830%0Ahttp://www.pubmedcentral.nih.gov/articlerender.fcgi?artid=PMC5751129 10.3390/ijms18122526 PMC5751129 29186830

[14] Murray C, Mann DL, Gerber LN, Barth W, Perlmann S, Decker JL, et al. Histocompatibility alloantigens in psoriasis and psoriatic arthritis. Evidence for the influence of multiple genes in the major histocompatibility complex. J Clin Invest. 1980;66(4):670–5. 10.1172/JCI109903 PMC371640 6932404

[15] Jordan CT, Cao L, Roberson EDO, Pierson KC, Yang CF, Joyce CE (2012). PSORS2 is due to mutations in CARD14. Am J Hum Genet.

[16] Husted JA, Gladman DD, Long JA, Farewell VT (1995). A modified version of the health assessment questionnaire (HAQ) for psoriatic arthritis. Clin Exp Rheumatol.

[17] Kane D, Stafford L, Bresniham B, FitzGerard O (2003). A prospective, clinical and radiological study of early psoriatic arthritis: an early synovitis clinic experience. Rheumatology.

[18] FitzGerald O, Haroon M, Giles JT, Winchester R. Concepts of pathogenesis in psoriatic arthritis: genotype determines clinical phenotype. Arthritis Res Ther. 2015;17(1). 10.1186/s13075-015-0640-3 PMC4422545 25948071

[19] Haroon M, Winchester R, Giles JT, Heffernan E, FitzGerald O (2016). Certain class I HLA alleles and haplotypes implicated in susceptibility play a role in determining specific features of the psoriatic arthritis phenotype. Ann Rheum Dis.

[20] •• Haroon M, Winchester R, Giles JT, Heffernan E, FitzGerald O. Certain class I HLA alleles and haplotypes implicated in susceptibility play a role in determining specific features of the psoriatic arthritis phenotype. Ann. Rheum. Dis. 2016b;75(1):155–62 **This paper identifies numerous genotype-phenotype correlations of HLA alleles with clinical and radiographic subtypes of PsA****.** 10.1136/annrheumdis-2014-205461 25261574

[21] Ho PYPC, Barton A, Worthington J, Thomson W, Silman AJ, Bruce IN (2007). HLA-Cw6 and HLA-DRB1 *07 together are associated with less severe joint disease in psoriatic arthritis. Ann Rheum Dis.

[22] Gladman DD, Kung TN, Siannis F, Pellett F, Farewell VT, Lee P (2005). HLA markers for susceptibility and expression in scleroderma. J Rheumatol.

[23] Korendowych E, Dixey J, Cox B, Jones S, McHugh N (2003). The influence of the HLA-DRB1 rheumatoid arthritis shared epitope on the clinical characteristics and radiological outcome of psoriatic arthritis. J Rheumatol.

[24] Rahman P, Snelgrove T, Peddle L, Siannis F, Farewell V, Schentag C, et al. A variant of the IL4 I50V single-nucleotide polymorphism is associated with erosive joint disease in psoriatic arthritis. Arthritis Rheum. 2008;58(7):2207–8. 10.1002/art.23558 18576348

[25] Rahmati S, Abovsky M, Pastrello C, Kotlyar M, Lu R, Cumbaa CA, et al. pathDIP 4: an extended pathway annotations and enrichment analysis resource for human, model organisms and domesticated species. Nucleic Acids Res. [Internet]. 2019; Available from: http://www.ncbi.nlm.nih.gov/pubmed/31733064. 10.1093/nar/gkz989 PMC7145646 31733064

[26] Ahn R, Gupta R, Lai K, Chopra N, Arron ST, Liao W. Network analysis of psoriasis reveals biological pathways and roles for coding and long non-coding RNAs. BMC Genomics. 2016;17(1). 10.1186/s12864-016-3188-y PMC5084355 27793094

[27] Hiebert P, Werner S (2018). Targeting metabolism to treat psoriasis. Nat Med.

[28] Hosaka Y, Saito T, Sugita S, Hikata T, Kobayashi H, Fukai A (2013). Notch signaling in chondrocytes modulates endochondral ossification and osteoarthritis development. Proc. Natl. Acad. Sci U. S. A. National Academy of Sciences.

[29] Ota T, Takekoshi S, Takagi T, Kitatani K, Toriumi K, Kojima T, et al. Notch signaling may be involved in the abnormal differentiation of epidermal keratinocytes in psoriasis. Acta Histochem Cytochem. 2014;47(4):175–83. 10.1267/ahc.14027 PMC4164705 25392571

[30] Skarmoutsou E, Trovato C, Granata M, Rossi GA, Mosca A, Longo V, et al. Biological therapy induces expression changes in Notch pathway in psoriasis. Arch Dermatol Res. 2015;307(10):863–73. 10.1007/s00403-015-1594-7 26319047

[31] Gudjonsson JE, Johnston A, Stoll SW, Riblett MB, Xing X, Kochkodan JJ, et al. Evidence for altered wnt signaling in psoriatic skin. J Invest Dermatol. 2010;130(7):1849–59. 10.1038/jid.2010.67 PMC2886156 20376066

[32] M.T. Patrick, P.E. Stuart, K. Raja, S. Chi, Z. He, J.J. Voorhees, et al. Integrative approach to reveal cell type specificity and gene candidates for psoriatic arthritis outside the MHC. Front. Genet. [Internet]. 2019a;10(APR). Available from: 10.3389/fgene.2019.00304 PMC6470186 31031798

[33] •• M.T. Patrick, P.E. Stuart, K. Raja, S. Chi, Z. He, J.J. Voorhees,et al. Integrative approach to reveal cell type specificity and gene candidates for psoriatic arthritis outside the MHC. Front. Genet. [Internet]. 2019b;10(APR). Available from: 10.3389/fgene.2019.00304. **This article reveals epigenomic elements that can provide useful insights from suggestive susceptibilty loci from GWAS studies.** PMC6470186 31031798

[34] Mavropoulos A, Rigopoulou EI, Liaskos C, Bogdanos DP, Sakkas LI. The role of p38 mapk in the aetiopathogenesis of psoriasis and psoriatic arthritis. Clin Dev Immunol. 2013;2013. 10.1155/2013/569751 PMC3787653 24151518

[35] Zhao W, Xiao S, Li H, Zheng T, Huang J, Hu R, et al. MAPK phosphatase-1 deficiency exacerbates the severity of imiquimod-induced psoriasiform skin disease. Front. Immunol. 2018. 10.3389/fimmu.2018.00569 PMC5873221 29619028

[36] Zheng T, Zhao W, Li H, Xiao S, Hu R, Han M, et al. P38α signaling in Langerhans cells promotes the development of IL-17-producing T cells and psoriasiform skin inflammation. Sci. Signal. 2018;11(521). 10.1126/scisignal.aao1685 29535261

[37] Chimenti MS, Sunzini F, Fiorucci L, Botti E, Fonti GL, Conigliaro P (2018). Potential role of cytochrome c and Tryptase in psoriasis and psoriatic arthritis pathogenesis: focus on resistance to apoptosis and oxidative stress. Front Immunol.

[38] Kastelan M, Prpić-Massari L, Brajac I. Apoptosis in psoriasis. Acta Dermatovenerol. Croat. [internet]. 2009;17(3):182–6. Available from: http://www.ncbi.nlm.nih.gov/pubmed/19818217. 19818217

[39] Weatherhead SC, Farr PM, Jamieson D, Hallinan JS, Lloyd JJ, Wipat A, et al. Keratinocyte apoptosis in epidermal remodeling and clearance of psoriasis induced by UV radiation. J Invest Dermatol. 2011;131(9):1916–26. 10.1038/jid.2011.134 PMC3160491 21614017

[40] Neumann E, Khawaja K, Müller-Ladner U. G protein-coupled receptors in rheumatology. Nat. Rev. Rheumatol. [internet]. 2014;10(7):429–436. Available from: http://www.ncbi.nlm.nih.gov/pubmed/24798574. 10.1038/nrrheum.2014.62 24798574

[41] Coates LC, Helliwell PS (2017). Psoriatic arthritis: state of the art review. Clin. Med. J. R. Coll. Physicians London.

[42] Fort JG, Smith JB, Abruzzo JL (1993). Abnormal T-cell function in patients with psoriatic arthritis: evidence for decreased interleukin 2 production. Rheumatol Int.

[43] Kang EJ, Kavanaugh A (2015). Psoriatic arthritis: latest treatments and their place in therapy. Ther Adv Chronic Dis.

[44] Yan D, Afifi L, Jeon C, Trivedi M, Chang HW, Lee K (2017). The metabolomics of psoriatic disease. Psoriasis Targets Ther.

[45] Rahmati S, Abovsky M, Pastrello C, Jurisica I. pathDIP: an annotated resource for known and predicted human gene-pathway associations and pathway enrichment analysis. Nucleic Acids Res. [Internet]. 2017 Jan [cited 2017 Apr 28];45(Database issue):D419–D426. Available from: http://www.ncbi.nlm.nih.gov/pmc/articles/PMC5210562/10.1093/nar/gkw1082 PMC5210562 27899558

[46] King KE, George AL, Sakakibara N, Mahmood K, Moses MA, Weinberg WC (2019). Intersection of the p63 and NF-κB pathways in epithelial homeostasis and disease. Mol Carcinog.

[47] Shamilov R, Aneskievich BJ. TNIP1 in autoimmune diseases: regulation of toll-like receptor signaling. J Immunol Res. 2018;2018. 10.1155/2018/3491269 PMC6192141 30402506

[48] Bateman A (2019). UniProt: a worldwide hub of protein knowledge. Nucleic Acids Res.

[49] Blake JA, Christie KR, Dolan ME, Drabkin HJ, Hill DP, Ni L, et al. Gene ontology consortium: Going forward. Nucleic Acids Res. [Internet]. 2015 Jan [cited 2016 Jan 19];43(D1):D1049–56. Available from: http://nar.oxfordjournals.org/lookup/doi/10.1093/nar/gku1179 10.1093/nar/gku1179 PMC4383973 25428369

[50] Patrick MT, Stuart PE, Raja K, Gudjonsson JE, Tejasvi T, Yang J, et al. Genetic signature to provide robust risk assessment of psoriatic arthritis development in psoriasis patients. Nat. Commun. 2018a;9(1). 10.1038/s41467-018-06672-6 PMC6177414 30301895

[51] Stuart PE, Nair RP, Tsoi LC, Tejasvi T, Das S, Kang HM, et al. Genome-wide association analysis of psoriatic arthritis and cutaneous psoriasis reveals differences in their genetic architecture. Am J Hum Genet. 2015a;97(6):816–36. 10.1016/j.ajhg.2015.10.019 PMC4678416 26626624

[52] •• Patrick MT, Stuart PE, Raja K, Gudjonsson JE, Tejasvi T, Yang J, et al. Genetic signature to provide robust risk assessment of psoriatic arthritis development in psoriasis patients. Nat. Commun. 2018b;9(1) **Using machine learning methodology differences in genetic architecture between psoriasis subtypes were interrogated to develop risk assessment models for PsA.** 10.1038/s41467-018-06672-6 PMC6177414 30301895

[53] •• Stuart PE, Nair RP, Tsoi LC, Tejasvi T, Das S, Kang HM, et al. Genome-wide association analysis of psoriatic arthritis and cutaneous psoriasis reveals differences in their genetic architecture. Am. J. Hum. Genet. 2015b;97(6):816–36 **PsA GWAS followed by meta-analysis of other large datasets identified multiple significant loci and differences in various genetic loci were noted between cutanous psoriasis and PsA****.** 10.1016/j.ajhg.2015.10.019 PMC4678416 26626624

